# Structural Differences between the Lignin-Carbohydrate Complexes (LCCs) from 2- and 24-Month-Old Bamboo (*Neosinocalamus affinis*)

**DOI:** 10.3390/ijms19010001

**Published:** 2017-12-21

**Authors:** Pan-Pan Yue, Ya-Jie Hu, Gen-Que Fu, Chang-Xia Sun, Ming-Fei Li, Feng Peng, Run-Cang Sun

**Affiliations:** 1 Beijing Key Laboratory of Lignocellulosic Chemistry, Beijing Forestry University, Beijing 100083, China; ypp1109@bjfu.edu.cn (P.-P.Y.); huyajie@bjfu.edu.cn (Y.-J.H.); fugenque@bjfu.edu.cn (G.-Q.F.); limingfei@bjfu.edu.cn (M.-F.L.); rcsun3@bjfu.edu.cn (R.-C.S.); 2 College of Science, Beijing Forestry University, Beijing 100083, China; sxinghan@163.com

**Keywords:** 2- and 24-month-old bamboo, chemical composition, LCC preparations, lignin and lignin–carbohydrate complex linkages, 2D-HSQC NMR

## Abstract

The lignin-carbohydrate complex (LCC) was isolated from milled wood lignin of 2- and 24-month-old crude bamboo (*Neosinocalamus affinis*) culms using acetic acid (AcOH) and then characterized. The results have shown that the LCC preparation from 2-month-old bamboo (L_2_) exhibited a slightly lower molecular weight than the LCC preparation from the 24-month-old bamboo (L_24_). Further studies using Fourier transform infrared spectroscopy (FT-IR) and heteronuclear single quantum coherence (2D-HSQC) NMR spectra analyses indicate that the LCC preparations included glucuronoarabinoxylan and G-S-H lignin-type with G>S>>H. The content of the S lignin units of LCC in the mature bamboo was always higher than in the young bamboo. Combined with sugar composition analysis, the contents of phenyl glycoside and ether linkages in the L_24_ preparation were higher than in the L_2_ preparation; however, there was a reverse relationship of ester LCC bonds in L_2_ and L_24_. Lignin–xylan was the main type of LCC linkage in bamboo LCCs. Lignin–lignin linkages in the LCC preparations included β-β, β-5 and β-1 carbon-to-carbon, as well as β-*O*-4 ether linkages, but β-1 linkages were not present in L_2_.

## 1. Introduction

Bamboo, an abundant renewable biomass resource, consists of cellulose, hemicelluloses, and lignin, which are associated with one another through physical binding and chemical linkages to form complex structures [1,2]. Based on the current state of knowledge, cellulose is a linear homopolysaccharide, which is composed of D-glucose units linked by β-(1–4) glycosidic bonds [3]. Hemicelluloses are polysaccharides, which include xylans, mannans, β-glucans with mixed linkages, and xyloglucans [4]. Lignin consists of phenylpropane units of the guaiacyl (G), syringyl (S), and *p*-hydroxyphenyl (H) units, which are connected with a relatively low amount of *p*-coumarate and ferulic esters of lignin, and the inter-units of lignin are mainly linked by chemical bonds of β-*O*-4, β-β, β-5, and β-1 types [5,6]. Lignin in the S_2_ layer of the secondary wall has a greater chance of having covalent or hydrogen bonds with carbohydrates [7]. In isolated samples, most lignin is attached to hemicelluloses but not to cellulose, probably because cellulose forms strings that are partly crystalline [8]. Lignin and hemicelluloses are generally considered to be linked to one another through covalent bonds, which gives them high mechanical strength and makes the plant cell walls resistant to biodegradation. These bonds and interactions are thought to interfere with their separation from and the degradation of the plant [9]. This may be crucial to the rigidity of lignified plant cell walls.

It is generally supposed that lignin polymers are linked covalently to carbohydrates to form lignin–carbohydrate complexes (LCCs) [8]. Covalent linkages between lignin and carbohydrates have been found in wood; however, knowledge of its types, frequencies, and quantities are still insufficient [10]. Three main types of LCC linkages in the lignocellulosic biomass include benzyl esters, benzyl ethers, and phenyl glycosides. Extensive investigations have been carried out to separate and elucidate the nature of LCC, and there have been some comprehensive evaluations of the subject [11,12]. Imamura et al. [13] reported that Japanese beech water-soluble LCC has a 7.6% lignin content, an 80% carbohydrate content, and a 10% uronic acid content. Karlsson and Westermark [14] used the LiCl–dimethylacetamide cellulose solvent system to dissolve kraft pulps prepared from pine and birch and found that a considerable amount of the residual lignin was chemically linked to the high molecular weight cellulose in pine but not in birch. Yaku et al. [15] studied for the first time that acidic LCCs separated from pine Björkman LCCs form micelles. Although some research about the chemical and physical properties of LCCs has been conducted, there is little evidence about the chemical composition and structure of bamboo (*Neosinocalamus affinis*) LCCs. More importantly, the characteristics of LCC linkages have not yet been completely elucidated due to its structural complexity. 

The isolation of LCCs is an essential step in analyzing cell–wall compounds. Therefore, to obtain dependable and representative results, the method that is used to prepare the LCCs is a critical factor. The methods used for isolating LCCs include water, organic solvent, and water-organic solvent extractions. The structures of the LCCs prepared from different isolation methods present similarities and differences [16,17]. In this paper, the LCC preparations were obtained by purifying milled wood lignin (MWL) from crude bamboo culms with AcOH. Azuma et al. [18] have reported on the LCC extracted from the milled wood lignin from *Pinus densiflora*; the main backbone structure of the carbohydrate moiety in the LCC fractions consisted of (l→4)-linked d-mannopyranosyl residues with high branches. The investigation by Freudenberg et al. [19] has shown that the LCC bonds are divided into α-ether, phenyl glycoside, acetal, ester bonds, and combination types for -C-O- or -C-C- of free radical linkages. There are many studies on the chemical bond linkages of LCCs; however, knowledge about LCC linkages is still uncertain. 

In the bamboo cell wall, polyphenol-ferulic acid (FA) is linked by an ester bond to the hemicellulose, which is connected to the lignin with an ether bond, forming a structure with hemicellulose-ester-ferulic acid-ether-lignin linkages. The cell walls from bamboo with different ages and from different species will have different chemical compositions; this is the basis for utilizing bamboo to research diversity. At present, there is a great deal of knowledge about wood; however, little information is available on the composition of LCC in bamboo. Basically, studies on LCC formation and structure during the growth stages of bamboo is still limited. There are barely any studies on the structural characterization of LCC from *Neosinocalamus affinis* bamboo at different growth ages. According to our previous research [20], LCC preparations were isolated from 2-, 4-, and 6-month-old *Neosinocalamus affinis* bamboo to study the variation of bamboo chemical linkages during its early stages of development. The results showed that the amounts of β-5 and phenyl glycoside linkages increase with maturation of the bamboo, while the amounts of β-*O*-4, benzyl ether, and benzyl ester linkages first increase and then decrease with the development of the young bamboo culms [20]. Based on the previous research, the aim of this study was to explore structural differences in the lignin–carbohydrate complex (LCC) from young and mature bamboo (*Neosinocalamus affinis*). Thus, in this paper, LCC preparations were extracted from 2-month-old and 24-month-old *Neosinocalamus affinis* bamboo, which represent to some extent young and mature bamboo, respectively. The results have been compared for chemical composition, chemical structure, and chemical bond linkages. The structural characterization was determined by a variety of methods, including high-performance anion exchange chromatography (HPAEC), gel permeation chromatography (GPC), Fourier transform infrared (FT-IR), and heteronuclear single quantum coherence (HSQC) NMR. 

## 2. Results and Discussion

### 2.1. Chemical Composition of the Lignin-Carbohydrate Complex (LCC) Analysis

Table 1 summarizes the main chemical compositions of the LCC preparations. LCC fractions were subjected to carbohydrate and lignin analysis. Information relating to bamboo’s LCCs is still limited since the LCCs are difficult to separate and degrade as a result of the cross-linking between the lignin and the carbohydrates. The yield of LCC preparations was relatively low. The LCC preparations from 2-month-old (L_2_) and 24-month-old (L_24_) bamboo were 3.9% and 1.5% relative to the oven-dry weight of the ball-milled bamboo meal; the L_2_ and L_24_ were representative in this research. The LCC yield reduction was probably because a significant amount of lignin was isolated from the secondary walls, and under the same conditions the LCC preparation from mature bamboo was not easily extracted by the neutral solvent (96% dioxane). Classically, the chemical cross-linking between cell wall components is more robust with plant growth. The amounts of acid-soluble lignin (ASL) and acid-insoluble lignin (AIL) mostly represent the total lignin material. The acid-insoluble lignin was usually the dominant lignin component in the plant cell wall. The content of the acid-insoluble lignin in the L_2_ preparation was significantly lower than in the L_24_ preparation, while the reverse relationship was observed in the content of acid-soluble lignin. The result shows that the percentage of acid-soluble lignin of LCC in young bamboo culms was relatively higher than in the mature bamboo culms, similar to what was reported by our group [20]. This was likely caused by the release of more acid-soluble lignin from young bamboo samples extracted by the dioxane solution. This could be explained by the fact that the lignin structure in 2-month-old bamboo, which is tender and without fully formed bamboo culm, may be different to the mature bamboo. Besides, LCC preparations contained 28.0–39.4% carbohydrates. To verify the LCC linkages in bamboo, the sugar composition of the LCC preparations and the dimethyl sulfoxide (DMSO)-soluble hemicelluloses are summarized in Table 1 and Table 2. Glucose (72.1–39.8%) and xylose (18.7–52.2%) were the dominant monosaccharides, while only a small amount of arabinose, mannose, and galactose was detected in the LCC preparations. However, the L_2_ preparation showed a relatively higher amount of glucose (72.1%), which was approximately two-fold higher than in the L_24_ preparation. The strong enrichment of glucose units in the LCC preparation implies that the L_2_ preparation probably contained more glucan-type hemicelluloses [21]. Eriksson et al. [12] reported that there was no direct evidence for covalent linkages between lignin and cellulose.

The data in Table 2 clearly illustrates that the DMSO-soluble hemicelluloses were a mixture of many different monosaccharides. It is quite obvious that xylose was the main sugar of the 2-month-old bamboo hemicellulose (H_2_) and 24-month-old bamboo hemicellulose (H_24_) preparations, while trace amounts of arabinose, galactose, glucose (except for H_2_), and glucuronic acid were also identified in bamboo hemicelluloses. This demonstrates that the bamboo hemicelluloses consists mainly of glucuronoarabinoxylans; the α-Ara*f* and α-d-glucopyranosyl uronic units constitute the side chain of xylan. This result was in accordance with the analysis reported by Yoshida et al. [22]. The ratios of glucuronic acid to xylose (GlcA/Xyl) and arabinose to xylose (Ara/Xyl) represent the degree of linearity or branching of the hemicelluloses; a higher degree of branching of the xylan chains leads to a higher solubility of the polysaccharides [23]. In addition, the high amounts of glucose (22.01%) probably resulted from the β-d-glucopyranoside unit of glucan [24].

### 2.2. Fourier Transform Infrared (FT-IR) Spectra Analysis

**Figure 1:**
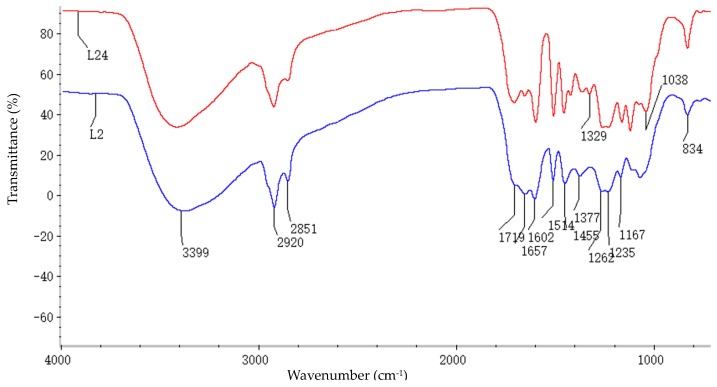
FT-IR of the lignin-carbohydrate complex (LCC) preparations (L_2_ and L_24_).

FT-IR spectra of L_2_ and L_24_ preparations are shown in Figure 1, and the main assignments of FT-IR signals are summarized in Table 3. The peak at 3399 cm^−1^ originated from the O–H stretching vibration in the aliphatic and aromatic OH groups, and the absorption bands in 2920 and 2851 cm^−1^ are due to C–H (CH_2_ and CH_3_) stretching vibrations. The absorptions at 1657 and 1719 cm^−1^ are attributed to carbonyl groups, but a peak is not observed at about 2730 cm^−1^, implying it is a ketone group rather than an aldehyde group. Signals ranging from 1500–1600 cm^−1^ are assigned to aromatic skeletal ring vibration in lignin. The peaks at 1514 and 1455 cm^−1^ are not to be overlapped with the bands of carbohydrates, and they represent the absorption of lignin to an extent. The peaks at 1262 cm^−1^, 1329 cm^−1^, and 1167 cm^−1^ seem to be due to guaiacyl-, syringyl-, and *p*-hydroxyphenyl units of lignin moieties, respectively [25]. From the intensity of the absorbance at 1329 cm^−1^, L_24_ has a relatively stronger absorption than L_2_, which indicates that there was almost no syringyl in the 2-month-old bamboo LCC preparation and that the amounts of syringyl types increase with the increasing maturity of the bamboo. The C–O, C–C, and C=O stretching vibrational mode of the aromatic skeletal ring of the guaiacyl type is recognized by the presence of a signal at 1235 cm^−1^. In addition, the intense signal at 834 cm^−1^ is assigned to the aromatic C–H out-of-plane vibrations in the H unit and C–H out-of-plane vibrations in the 2, 5, and 6 positions in the G units, respectively [26,27]. 

### 2.3. HSQC NMR Spectra Analysis

**Figure 2:**
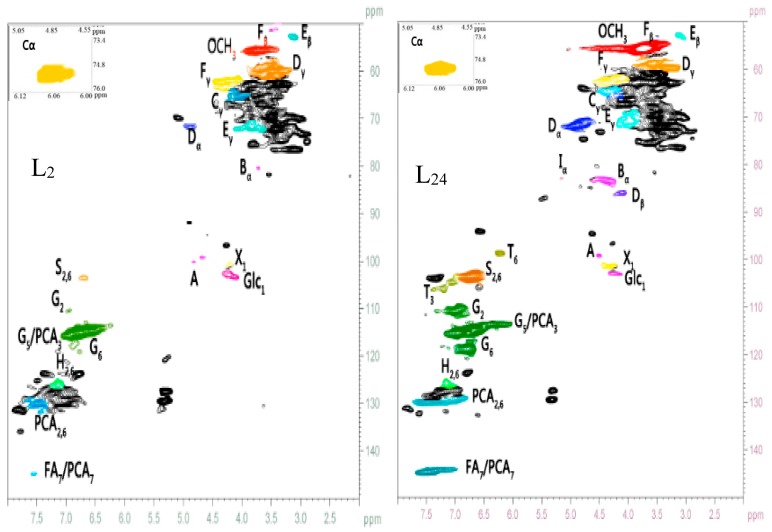
Main classical structures of LCC preparations identified by 2D-HSQC spectra: (A) phenyl glycoside; (B) benzyl ether; (C) ester (C_α_, α-ester; C_γ_, γ-ester); (D) β-*O*-4 substructure; (E) β-β resinol substructure; (F) β-5 phenylcoumaran substructure; (I) β-1 spirodienone substructure; (G) guaiacyl unit; (H) *p*-hydroxyphenyl unit; (S) syringyl unit; (T) tricin; (PCA) *p*-coumarate; (FA) ferulic acid unit; (Glc) β-d-glucopyranoside unit (X) β-d-xylopyranoside unit. Different colors represent different structure units.

**Figure 3:**
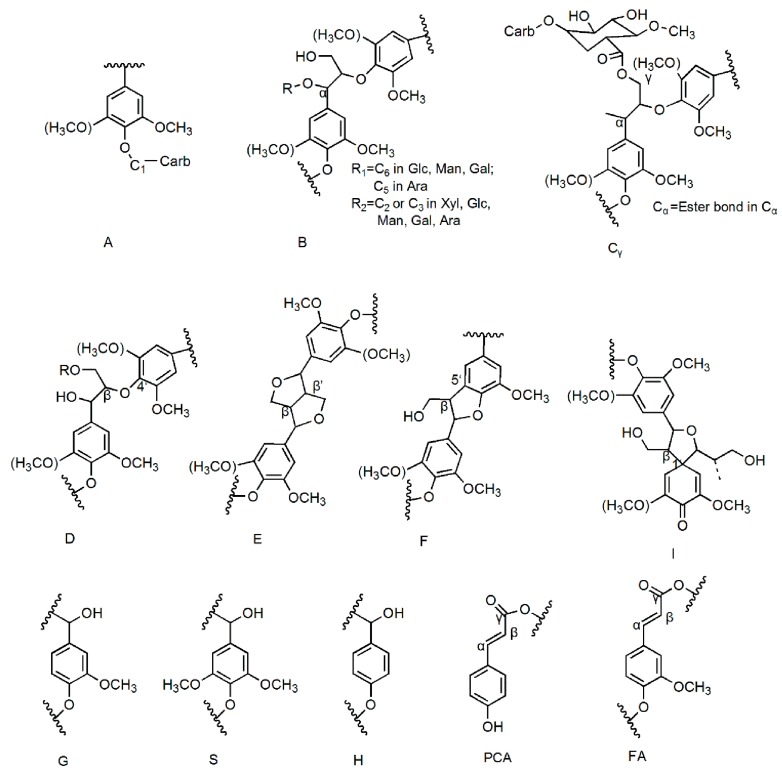
Main structures present in the LCC preparations: (A) phenyl glycoside; (B) benzyl ether; (C) ester (C_α_, α-ester; C_γ_, γ-ester); (D) β-*O*-4 substructure; (E) β-β resinol substructure; (F) β-5 phenylcoumaran substructure; (I) β-1 spirodienone substructure; (G) guaiacyl unit; (S) syringyl unit; (PCA) *p*-coumarate; (H) *p*-hydroxyphenyl unit; (FA) ferulate unit.

The methods of wet chemistry techniques and model experiments are useful for acquiring information, while direct observation of LCC structure with spectroscopic techniques is also of primary importance [28]. NMR spectroscopy is extremely useful in ascertaining the main linkages of lignin–carbohydrate. The compositions of lignin and carbohydrates are quite complex, and thus it is difficult to distinguish the linkages of lignin and carbohydrate by general ^13^C-NMR. However, 2D-HSQC can distinguish the overlapping signals presented in the ^1^H and ^13^C spectra, and the signal assignment is also better than in the case of 1D NMR; thus, it provides crucial structural information of the sample [29]. Classically, the 2D-HSQC spectra of lignin samples can be divided into three regions of ^13^C–^1^H correlations corresponding to the aliphatic (δ_C_/δ_H_ 10–40/0.5–2.5), side chain (δ_C_/δ_H_ 50–95/2.5–6.0), and aromatic (δ_C_/δ_H_ 95–150/5.5–8.0) regions [30]. In this study, the L_2_ and L_24_ preparations extracted from 2-month-old and 24-month-old bamboo (*Neosinocalamus affinis*) were subjected to 2D-HSQC spectra analysis to obtain detailed information (Figure 2). The assignments of the main cross-signals in the HSQC spectra are presented in Table 4, and the corresponding LCC linkages and lignin substructures are listed in Figure 3.

#### 2.3.1. Lignin Side Chain and Aromatic Regions

The major inter-unit linkages within the lignin monomer (G, S, and H units) are β–β, β–5, and β–1 carbon-to-carbon as well as β–*O*–4 ether substructures. The side chain region (δ_C_/δ_H_ 50–95/2.5–6.0) from the 2D-HSQC spectra shows crucial information for the inter-unit linkages in lignin (Figure 2 and Table 4). The bamboo LCC preparation had structural complexity and the lignin linkages seem to be useful for acquiring information about the structure of LCCs. The β–*O*–4 linkages are the main inter-unit linkages in lignin [31]. The β–*O*–4 aryl ether linkages of L_2_ and L_24_ are presented at about δ_C_/δ_H_ 71.29/4.88 and 60.73/3.39 ppm, which correspond to resonances of C-α and C-γ positions of β–*O*–4 linkages, respectively. The C_β_–H_β_ correlation corresponding to the erythro forms of the S type β–*O*–4 substructures is presented at δ_C_/δ_H_ 83.56/4.24 ppm [32]. However, this signal disappeared in the side chain region of L_2_. In addition, the intense signals show the presence of β–*O*–4 in L_24_, while the corresponding cross-signals of L_2_ show relatively low intensities. The carbon–carbon linkages in the lignin structural units make it undegradable, and in the process of hydrogenated decomposition and alcoholysis lignin was not decomposed into a single unit due to the presence of carbon–carbon linkages. In addition, the common carbon–carbon linkages, such as the signals for β–β resinol substructures (C_β_–H_β_ in β–β units, 53.57/3.39 ppm; C_γ_–H_γ_ in β–β units, 70.26/3.96 ppm) and phenylcoumaran substructures (β–5), are observed in lower amounts in the preparations, and the signals for C_β_–H_β_ and C_γ_–H_γ_ correlations in β–5 units are observed at δ_C_/δ_H_ 53.57/3.39 and 62.92/3.96, respectively [32]. The C_α_–H_α_ correlation corresponding to the β–1 substructures in L_24_ is presented at δ_C_/δ_H_ 82.39/5.08. Small signals corresponding to β–1 are observed in the side chain region of L_24_ and disappeared in the side chain region of L_2._ All of these results imply that the amount of lignin structures in the L_2_ preparation was less than in the L_24_ preparation.

In the aromatic regions, cross-signals are observed from the aromatic phenyl rings of guaiacyl (G), *p*-hydroxyphenyl (H), and syringyl (S). The cross-signals at δ_C_/δ_H_ 115.89/6.76, 119.34/6.76, and 111.82/6.97 are attributed to C_5_–H_5_, C_6_–H_6_, and C_2_-H_2_ of G units, respectively. The S lignin units show the main cross peaks C_2,6_–H_2,6_ at δ_C_/δ_H_ 104.74/6.67; however, the signal strength in L_2_ was weaker than in L_24_, which is in agreement with the results obtained in the FT-IR spectra analysis. In addition, the S/G ratios are estimated to be 0.18 and 0.89 for L_2_ and L_24_ preparations, respectively (Table 4), which indicate that the S lignin units in mature bamboo are always higher than in young bamboo. This result is in good agreement with the research reported by Zhang et al. [20]. An important feature of lignin in all plant cell walls is that the S/G ratio can reflect, to a certain degree, the capacity to remove lignin. The C_2,6_–H_2,6_ correlations of the H units are observed as a weaker signal at δ_C_/δ_H_ 128.22/7.19, indicating that there were small amounts of H lignin units. This result is in accordance with the studies of Bai et al. [26] and Kang et al. [27]. Moreover, *p*-coumaric acid (PCA) and ferulic acid (FA) are observed in the 2D-HSQC spectra of the L_2_ and L_24_ preparations. The cross-peaks corresponding to C_7_–H_7,_ C_3_–H_3_, and C_2,6_–H_2,6_, δ_C_/δ_H_ at 144.76/7.43 (the signals of FA and PCA were overlapped), 115.69/6.76, and 130.11/7.43, respectively, are shown in Figure 2. 

The spectra of the LCC preparations exhibit mainly carbohydrate cross-signals (Figure 2), including β–d–xylopyranoside units (X_1_, X_2_, X_3_, and X_4_) and glucopyranoside units (Glc), which are overlapped with some other unassigned lignin moieties [33]. In addition, tricin is considered a main flavone in cereal crop plants, which is found mainly in the leaves and stems [34]. Cross-signals corresponding to C_3_–H_3_ at δ_C_/δ_H_ 106.39/7.19 and C_6_–H_6_ at δ_C_/δ_H_ 99.82/6.22 are shown in the L_24_ preparation 2D-HSQC spectra (Figure 2 and Table 4), which are attributed to tricin [26]. However, the above signals are not observed in the HSQC spectra of the L_2_ preparation. 

#### 2.3.2. Major LCC Linkages

The predominant types of LCC linkages consist of benzyl-ether, benzyl-ester, and phenyl-glycoside bonds [29,35]. Only limited research has focused on the quantitative information of these LCC linkages [28]. Some correlations in the anomeric regions may demonstrate the existence of phenyl glycoside linkages. It is noteworthy that phenyl glycoside linkages seem to be easily cleaved under mildly acidic conditions [36]. Phenyl glycoside linkages give the cross-signals of CH–1 at δ_C_/δ_H_ 104–99/5.2–4.8 in the 2D-HSQC spectrum; however, Figure 2 shows only one signal in the area of carbohydrates at δ_C_/δ_H_ 100.5/4.88, which is attributed to CH–1 included in the phenyl glycoside bonds based on data from corresponding model compounds [37]. This indicates that phenyl glycoside linkages are the bonds between carbohydrates (xylan and glucan) and lignin. Recently, Yuan et al. [33] reported that some lignin fractions with a high content of associated carbohydrates had been isolated. The lignin structure and LCC linkages have also been quantitated [38]. Analysis of carbohydrate compositions have shown that xylan is the main polysaccharide type in bamboo; thus, phenyl glycoside linkages consist mostly of the lignin-xylan type but also probably some lignin-glucan types (Table 1 and Table 2). 

The relative abundances of the main LCC inter-unit linkages and the molar S/G ratios of lignin in LCCs per 100 aromatic units (Ar) are illustrated in Table 5. According to semi-quantitative results, the amounts of phenyl glycoside linkages in L_2_ and L_24_ were 6.5 and 12.1 per 100Ar (monomeric lignin unit), respectively. Evidently, the amount of phenyl glycoside linkages in the L_24_ preparation was higher than in the L_2_ preparation. A significant amount of xylose was present in the hemicelluloses of the 24-month-old bamboo (Table 2), indicating that the lignin–xylan type of phenyl glycoside linkage was predominant in the mature bamboo LCC preparation. Zhang et al. [20] have recently studied phenyl glycoside linkages in bamboo LCC and the results match the data obtained in the present study fairly well. Other LCC linkages, such as ether linkages and ester LCC bonds, were also estimated by this methodology and the results are listed in Table 5.

Ether linkages are the primary type of alkali-stable LCC bonds [29]. Freudenberg et al. [19,39] have reported that the benzyl ether LCC bonds were estimated based on the model compound experiments. The cross-signals corresponding to the CH–α correlations of lignin and the primary OH groups of carbohydrates are observed in the area of 82.5–80.0/4.7–4.3 ppm (at C-6 of Glc, Gal, and Man, and C-5 of Ara) [40]. As shown in Table 5, the relative amount of benzyl ether LCC bonds in the L_2_ preparation (1.2/100 Ar) was lower than in the L_24_ preparation (1.9/100 Ar), and it was lower than the amount of phenyl glycoside linkages. Eriksson et al. [12] reported that some types of neutral sugar residues in hemicelluloses bind to lignin, probably by benzyl ether bonds. The data shown in Table 2 clearly illustrate that mature bamboo hemicelluloses contain more xylose because the L_24_ preparation had a higher content of benzyl ether bonds. In addition, C-6 of glucose is associated with lignin by benzyl ether LCC linkages [32]. Combined with sugar analysis, more glucose existed in H_2_, and thus the L_2_ preparation probably contained higher amounts of the lignin-glucan ether bond type. 

Ester LCC bonds are known as the primary type of alkali-labile LCC bond [29]. The signals for benzyl ester bonds at 75–77/6.0–6.2 ppm are attributed to the C_α_-H_α_ correlations. The C_γ_–H_γ_ signals in benzyl ester are observed in the 2D-HSQC spectrum at 65–62/4.5–4.0 ppm. The LCC linkages in bamboo are more sophisticated than in wood as a result of the ferulic acid and *p*-coumaric acid, and this will affect the calculation of the amount of benzyl ester. For this reason, the signal in area C_α_ (located in the left column) was integrated to estimate the amount of benzyl ester LCC bonds [20]. The signals at 72.74/3.37 and 73.89/3.48 ppm in the HSQC spectrum are assigned to CH_2_ and CH_3_ in glucuronic acid (GlcA), whereas the intensities of the cross-peak of glucuronic acid for L_24_ are weaker than that of L_2_ (Figure 2); thus, significant amounts of ester LCC bonds have been discovered in the L_2_ preparation. Table 5 shows that the relative amounts of benzyl ester LCC bonds in the L_2_ preparation (1.5/100 Ar) were higher than in the L_24_ preparation (0.5/100 Ar). Eriksson et al. [12] have reported that 4-*O*-methylglucuronic acid is linked to lignin by benzyl ester bonds. Taking the sugar analysis of hemicelluloses into consideration, the changes in the ester linkages are identical to the value of glucuronic acid.

### 2.4. Molecular Weight Analysis

The values of the weight-average (*M_w_*) and number-average (*M_n_*) molecular weights and the polydispersity (*M_w_/M_n_*) of L_2_ and L_24_ were determined by gel permeation chromatography (GPC) and the data are illustrated in Table 5. The weight-average molecular weights (*M_w_*) of L_2_ and L_24_ preparations were determined to be 8650 and 9890 g/mol, respectively. Clearly, the weight-average molecular weight of the L_24_ preparation was slightly higher than the L_2_ preparation. The L_2_ and L_24_ preparations exhibited similar polydispersity that was 1.10 and 1.02, respectively. Therefore, all LCC preparations had relatively narrow molecular weight distributions. The relatively low molecular weights of the LCC preparations suggest that the extract process degraded the LCC preparations to a noticeable extent. However, HSQC NMR and FT-IR spectra show that the process did not significantly change the core of the LCC structure.

## 3. Materials and Methods

### 3.1. Preparation of Bamboo Culms Powder

The bamboo (*Neosinocalamus affinis*) culms of 2-month-old and 24-month-old bamboo were harvested in Sichuan province, China, in winter. They were labeled as S_2_ and S_24_. The air-dried bamboo culms were cut into chips and were converted into bamboo meal (40–60 mesh particle size) in a micro plant grinding machine. Subsequently, the bamboo meals were extracted with a mixture of toluene/ethanol 2:1 (*v*/*v*) in a Soxhlet apparatus for 6 h. The sample of extractive-free meal, after drying over a cabinet oven at 60 °C, was ground in a planetary ball mill (Fritsch, Idar-Oberstein Germany) using a ZrO_2_ bowl with mixed balls to increase the accessibility of the substrate in subsequent experimental procedures. The 2-month-old ball-milled bamboo powder was darker than the 24-month-old ball-milled bamboo powder. All experiments were performed at least in duplicate.

### 3.2. Preparation of LCCs

**Figure 4:**
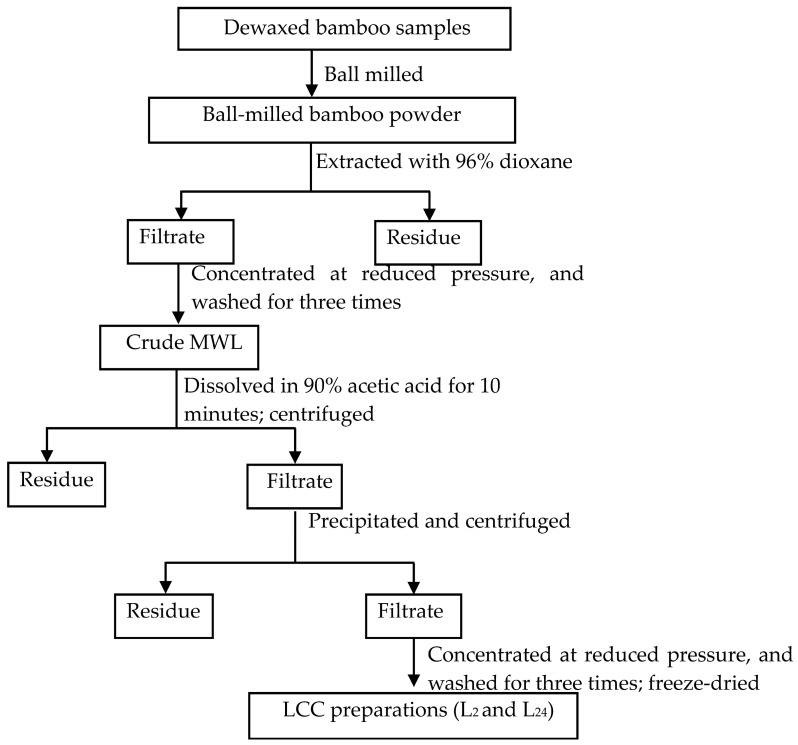
Scheme for LCC preparations (L_2_ and L_24_) isolated from 2-month-old and 24-month-old bamboo (*Neosinocalamus affinis*) crude milled wood lignin (MWL).

LCC preparations were isolated from 2-month-old and 24-month-old bamboo according to the methods of previous works [10,20]. The scheme is summarized in Figure 4, and the detailed procedures are as follows. The ball-milled sample was immersed in 96% aqueous dioxane (*v*/*v*) with a solid to liquid ratio of 1:20 (g/mL) at room temperature for 24 h under stirring. The procedure was conducted in the dark under a nitrogen atmosphere. Then, the residue was washed with aqueous dioxane, and the extracts and washings were collected. The residue was again extracted under the same conditions for three times. All of the extracts and washings were evaporated under vacuum at 50 °C, and a few drops of water were added to the solid matter and evaporated again. The milled wood lignin (MWL) was obtained. The crude MWL was dissolved in 90% acetic acid (*v*/*v*) and the precipitate that formed was separated by centrifugation and discarded. The solution was added dropwise with stirring to the deionized water. The precipitate that formed on addition of the deionized water was separated from the solution by centrifugation. The solution was concentrated by rotary vacuum evaporation and then freeze-dried to obtain LCC preparations (labeled as L_2_ and L_24_).

### 3.3. Preparation of Hemicelluloses

In order to better understand the structure of LCCs, the hemicelluloses were extracted from the delignification samples. According to the literature [24], the holocellulose was extracted by dimethyl sulfoxide (DMSO) with a solid to liquid ratio of 1:25 (g/mL) at 80 °C for 7 h under stirring. The filtrate was concentrated in a rotary evaporator at reduced pressure and precipitated in three volumes of ethanol under stirring. The precipitate was recovered by centrifugation (3500 rpm, 15 min) and freeze-drying; hemicelluloses were obtained (labeled as H_2_ and H_24_).

### 3.4. Analytical Methods

The chemical compositions of the bamboo culms LCCs were determined according to the standard of National Renewable Energy Laboratory (NREL) [41]. Molecular weights and molecular weight distributions of all LCC preparations were analyzed by gel permeation chromatography (GPC) using a PL-gel 10 μm Mixed-B 7.5 mm i.d. column. GPC analyses were run at least twice [32]. The LCC sample (4 mg) was dissolved in chromatographically pure tetrahydrofuran (2 mL), filtrated through a 0.22 μm organic filter, and then 10 μL solution was injected. The column was operated at ambient temperature and eluted with tetrahydrofuran at a flow rate of 1 mL/min. The hemicellulose fraction (4~6 mg) was hydrolyzed in 1 M sulphuric acid at 105 °C for 2.5 h. The composition of neutral sugars and uronic acid in the samples was analyzed by high-performance anion exchange chromatography (HPAEC) system (Dionex ISC 3000, Sunnyvale, CA, USA) [42]. All measurements were performed at least in duplicate.

The samples were prepared by grinding 1 mg sample/100 mg pre-dried KBr. Then they were determined in a FT-IR spectrometer with 64 scans between 4000 cm^−1^ and 400 cm^−1^ at 4 cm^−1^ resolution [43]. 2D-HSQC spectra were recorded by a Bruker NMR spectrometer at 400 MHz, using dimethyl sulfoxide-*d*_6_ (DMSO-*d*_6_) as the solvent. The amounts of LCC preparation linkages were calculated by the mean of parallel samples and the results were expressed as how many linkages per 100 aromatic rings [44]. The formulas are listed as follows:

(1) IC9 units=0.5IS2,6+IG2+0.5IH2,6 (grass lignin)

(2) AX=IX/IC9×100%

In the formula, all the integration should be in the same contour level. IS2,6, IG2, and IH2,6 represent the integration of S2,6, G2, and H2,6, respectively. AX and IC9 represent the integration of the amount of the main LCC linkages and the aromatic ring, where IX is the integral value of the objective linkages. Table 5 shows the calculation of the amounts of the LCC linkages and the S/G ratios of the LCC preparations.

## 4. Conclusions

Structural characterization of the lignin–carbohydrate complex (LCC) was conducted on 2-month-old and 24-month-old bamboo (*Neosinocalamus affinis*). It was found that the LCC preparations consisted of glucuronoarabinoxylan and the G-S-H lignin type with G>S>>H. The content of the S units in the LCC preparation from mature bamboo was always higher than from young bamboo. An analysis of the main chemical composition revealed that the content of the acid-soluble lignin in young bamboo LCC is higher than in mature bamboo LCC. The relatively low molecular weight of LCC preparations suggests that this process degraded the LCC to a degree, while 2D-HSQC NMR and FT-IR spectra show that the process did not strongly affect LCC primary structure. An analysis of the sugar composition and the 2D-HSQC NMR show that the content of phenyl glycoside and ether linkages in the L_24_ preparation was higher than in the L_2_ preparation, and that the reverse relationship was present for the ester LCC bonds in the L_2_ and L_24_ preparations. Lignin–xylan is the main type of LCC linkage in the bamboo LCC. Lignin–lignin linkages in the LCC preparations include β-β, β-5, and β-1 carbon-to-carbon as well as β-*O*-4 ether linkages, but the β-1 type was not present in L_2_. Our studies have provided evidence for differences in LCC structures between the cell walls from young and mature *Neosinocalamus affinis* bamboo culms.

## Figures and Tables

**Table 1:** Yield, chemical composition, and carbohydrate content of lignin-carbohydrate complex (LCC) preparations isolated from 2-month-old (L_2_) and 24-month-old (L_24_) bamboo (*Neosinocalamus affinis*).

Sample	Yield ^a^	Chemical Composition ^b^ (% of Relative Content)			Carbohydrate Content ^c^ (% of Relative Molar Content)				
		ASL	AIL	Carb	Ara	Gal	Glc	Xyl	Man
L_2_	3.9	51.9 ± 0.1	20.1 ± 0.3	28.0 ± 0.6	1.2 ± 0.1	5.0 ± 0.0	72.1 ± 0.3	18.7 ± 0.1	3.0 ± 0.0
L_24_	1.5	10.9 ± 0.3	49.7 ± 0.7	39.4 ± 0.4	4.2 ± 0.1	2.2 ± 0.2	39.8 ± 0.2	52.2 ± 0.2	1.6 ± 0.1

^a^ Relative to the oven-dry weight of the ball-milled bamboo powder (%); ^b^ ASL, acid-soluble lignin; AIL, acid-insoluble lignin; Carb, carbohydrates; ^c^ Ara, arabinose; Gal, galactose; Glc, glucose; Man, mannose; Xyl, xylose.

**Table 2:** Sugar composition of dimethyl sulfoxide (DMSO)-soluble hemicelluloses extracted from 2-month-old and 24-month-old bamboo (*Neosinocalamus affinis*) culms.

Sample ^a^	Molar Composition ^b^ (Relative %, mol/mol)					Molar Ratio ^c^	
	Ara	Gal	Glc	Xyl	GlcA	GlcA/Xyl	Ara/Xyl
H_2_	11.25 ± 0.2	4.60 ± 0.1	22.01 ± 0.1	58.30 ± 0.0	3.59 ± 0.1	0.06	0.19
H_24_	6.48 ± 0.1	0.59 ± 0.0	2.87 ± 0.2	86.93 ± 0.0	3.30 ± 0.0	0.03	0.07

^a^ H_2_ and H_24_ represent the 2-month-old and 24-month-old bamboo hemicelluloses, respectively. ^b^ Ara, arabinose; Gal, galactose; Glc, glucose; Xyl, xylose; GlcA, glucuronic acid. ^c^ Ara/Xyl, molar ratio of arabinose to xylose; GlcA/Xyl, molar ratio of glucuronic acid to xylose.

**Table 3:** Assignments of the FT-IR spectra of the LCC preparations.

Wave Numbers (cm^−1^)	Assignments
3399	O–H stretch
2920 and 2851	C–H stretch in methyl and methylene groups
1719	C=O stretch (unconjugated ketones, carbonyl and in ester groups in carbohydrate)
1657	Conjugated C=O stretch (lignin)
1602	Aromatic skeletal vibrations (lignin)
1514	Aromatic skeletal vibrations (lignin)
1455	Aromatic skeletal vibrations combined with C–H in-plane deform (lignin and methylene groups in polysaccharide)
1377	COO-asymmetric and symmetrical vibrations in carboxylate groups
1329	Syringyl units
1262	Guaiacyl units
1235	C–C, C–O, and C=O stretch of G ring
1167	Typical for HGS lignins; C=O in ester groups (conjugated)
1038	Aromatic C–H in-plane deformation, G > S; plus C–O deform, in primary alcohols; plus C=O stretch (unconjugated)
834	C–H out-of-plane in positions 2, 5, and 6 of G units

**Table 4:** Assignments of primary lignin and carbohydrate ^13^C–^1^H cross-signals in the HSQC spectra of the LCC preparations from bamboo (*Neosinocalamus affinis*).

Label	δ_C_/δ_H_ (ppm)	Assignments
-OCH_3_	56.09/3.72	C–H in methoxyls
S_2,6_	104.74/6.67	C_2,6_–H_2,6_ in syringyl units (S)
G_2_	111.82/6.97	C_2_–H_2_ in guaiacyl units (G)
G_5_	115.89/6.76	C_5_–H_5_ in guaiacyl units (G)
G_6_	119.34/6.76	C_6_–H_6_ in guaiacyl units (G)
H_2,6_	128.22/7.19	C_2,6_–H_2,6_ in *p*–hydroxyphenyl units (H)
D_α_	71.29/4.88	C_α_–H_α_ in β–*O*–4 structures linked to a S unit (D)
D_β_	83.56/4.24	C_β_–H_β_ in β–*O*–4 structures linked to G/H units (D)
D_γ_	60.73/3.39	C_γ_–H_γ_ in β–*O*–4 structures (D)
E_β_	52.97/3.09	C_β_–H_β_ in β–β structures (E)
E_γ_	70.26/3.96	C_γ_–H_γ_ in β–β structures (E)
F_β_	53.57/3.39	C_β_–H_β_ in β–5 structures (F)
F_γ_	62.92/3.96	C_γ_–H_γ_ in β–5 structures (F)
I_α_	82.39/5.08	C_α_–H_α_ in β–1 structures (I)
I_β_	59.62/2.72	C_β_–H_β_ in β–1 structures (I)
A	100.5/4.88	Phenyl glycoside linkages (A)
B_α_	82.5–80.0/4.7–4.3	C_α_–H_α_ in benzyl ether LCC bonds (B)
C_α_	77.0–75.0/6.2–6.0	α–Ester (C)
C_γ_	65–62/4.5–4.0	γ-Ester (C)
FA_7_/PCA_7_	144.76/7.43	C_7_–H_7_ in *p*-coumaroylated substructures (PCA) and ferulate acid (FA)
PCA_3_	115.69/6.76	C_3_–H_3_ in *p*-coumaroylated substructures (PCA)
PCA_2,6_	130.11/7.43	C_2,6_–H_2,6_ in *p*-coumaroylated substructures (PCA)
X2	72.74/3.09	C_2_–H_2_ in β–d–xylopyranoside (X)
X3	73.89/3.37	C_3_–H_3_ in β–d–xylopyranoside (X)
X4	75.38/3.40	C_4_–H_4_ in β–d–xylopyranoside (X)
GlcA_2_	72.74/3.37	C_2_–H_2_ in glucuronic acid (GlcA)
GlcA_3_	73.89/3.48	C_3_–H_3_ in glucuronic acid (GlcA)
X1	102.21/4.23	C_1_–H_1_ in β–d–xylopyranoside (X)
Glc_1_	103.20/4.20	C_1_–H_1_ in β–d–glucopyranoside (Glc)
T3	106.39/7.19	C_3_–H_3_ in tricin
T6	99.82/6.22	C_2,6_–H_2,6_ in tricin

**Table 5:** Quantification of LCC linkages (%, based on 100 Ar) and average molecular weight of the L_2_ and L_24_ preparations.

Sample	LCC Linkages ^a^				Average Molecular Weight (g/mol)		
	Phenyl Glycoside	Benzyl Ether	α-Ester	S/G ^b^	*M_w_*	*M_n_*	*M_w_/M_n_*
L_2_	6.5	1.2	1.5	0.18	8650	8140	1.06
L_24_	12.1	1.9	0.5	0.87	9890	9670	1.02

^a^ Per 100 Ar, pooled standard error (±5%); ^b^ S/G, syringyl/guaiacyl.
